# Self-organizing Maps and Bayesian Regularized Neural Network for Analyzing Gasoline and Diesel Price Drifts

**DOI:** 10.1007/s44196-021-00060-7

**Published:** 2022-01-03

**Authors:** R. Sujatha, Jyotir Moy Chatterjee, Ishaani Priyadarshini, Aboul Ella Hassanien, Abd Allah A. Mousa, Safar M. Alghamdi

**Affiliations:** 1 School of Information Technology and Engineering, Vellore Institute of Technology, Vellore, 632014 India; 2 Department of IT, Lord Buddha Education Foundation, Kathmandu, 44600 Nepal; 3 Scientific Research Group in Egypt (SRGE), Giza, 12613 Egypt; 4 Department of Electrical and Computer Engineering, University of Delaware, Newark, DE 19711 USA; 5 Faculty of Computers and Information, Cairo University, Giza, 12613 Egypt; 6 Department of Mathematics and Statistics, College of Science, Taif University, Taif, 21944 Saudi Arabia

**Keywords:** Self-organizing maps (SOM), Levenberg-Marquard (LM), Scaled conjugate gradient (SCG), Bayesian regularization (BR), Bayesian regularized neural networks (BRNNs), Nonlinear autoregressive neural network (NAR-NN)

## Abstract

Any nation’s growth depends on the trend of the price of fuel. The fuel price drifts have both direct and indirect impacts on a nation’s economy. Nation’s growth will be hampered due to the higher level of inflation prevailing in the oil industry. This paper proposed a method of analyzing Gasoline and Diesel Price Drifts based on Self-organizing Maps and Bayesian regularized neural networks. The US gasoline and diesel price timeline dataset is used to validate the proposed approach. In the dataset, all grades, regular, medium, and premium with conventional, reformulated, all formulation of gasoline combinations, and diesel pricing per gallon weekly from 1995 to January 2021, are considered. For the data visualization purpose, we have used self-organizing maps and analyzed them with a neural network algorithm. The nonlinear autoregressive neural network is adopted because of the time series dataset. Three training algorithms are adopted to train the neural networks: Levenberg-Marquard, scaled conjugate gradient, and Bayesian regularization. The results are hopeful and reveal the robustness of the proposed model. In the proposed approach, we have found Levenberg-Marquard error falls from − 0.1074 to 0.1424, scaled conjugate gradient error falls from − 0.1476 to 0.1618, and similarly, Bayesian regularization error falls in − 0.09854 to 0.09871, which showed that out of the three approaches considered, the Bayesian regularization gives better results.

## Introduction

The transportation industry has come a long way from horses and mules in the early days to railways, airlines, cruises, municipal transportation companies, cargo tracking, and express delivery services in today’s world. This industry finds its uses in moving people, animals, and goods by land, air, or sea, and as a global necessity, generates revenue worth billions of dollars. The transportation industry services majorly rely on the usage and pricing of gasoline and diesel, which are prone to fluctuations worldwide. The United States, being the most significant consumer, has recently witnessed price drifts concerning gasoline and diesel. The significant factors that influence these two components’ pricing are crude oil prices, processing and distribution costs, demand, taxation, currencies, and availability of local sources [1].

Moreover, the national pricing policy determines the cost paid by consumers. Between 2004 and 2008, the demand for crude oil went up, leading to increased gasoline costs. Prices jumped from $35 to $145 per barrel leading to an increase in gas costs. Gasoline prices in the United States (US) are also affected by fuel taxes as both federal and state taxes apply. Transporting crude oil to refineries and points of distribution as per demand incurs additional costs. Some other factors that contributed to gasoline cost could be in the form of extreme weather conditions, natural disasters in areas producing oil, legislation by many states for cleaner burning fuel, etc. [2]. US consumption of gasoline is seasonal because during summer, when people drive, the prices go up, whereas in winter, it reduces significantly [3]. However, over the last couple of years, the drift in gasoline and diesel costs has been attributed to many other reasons that were not seen before. As COVID-19 took the world by storm, economies were wrecked, and people stopped traveling [4, 5]. The lockdown imposed further led to lesser consumption of gasoline and diesel [6]. The average price in most states became higher than it was a year ago. Moreover, the brutal winter weather across many states in the US impacted oil production, which forced refineries to close in the top crude producing state. Many refineries were closed down, cutting off nearly 20% of the country’s refining capacity.

The sudden dip in demand led to many US oil and gas losing revenues worth millions of dollars and tens of thousands of jobs. As people started leaning on credit cards more than usual, oil companies suffered drastically. Many researchers and industry experts are hopeful that the capacity may come back online sometime during 2022–2023. As the lockdown impositions are taken down and more Americans are vaccinated, life may return to normal soon. It may witness people driving and flying more frequently, eventually increasing fuel costs, thereby ensuring some recovery. It may also be possible that people replace their vehicles with much more efficient models due to stimulus checks; this aid may lift gas prices if consumers spend it on the transportation industry [7]. Goldman Sachs estimates that $2 trillion in economic stimulus spending is predicted over 2021 and 2022, ultimately leading to an increased US oil demand by an estimated 200,000 barrels a day [8]. Late March 2020 witnessed that while most US was under lockdown orders, the national average astoundingly dropped down to $1.99 a gallon. It was found that many states had gas for less than $1 in April 2020. Based on all these statistics, we observe a fluctuation in the prices over the last few years due to various reasons, and hence there is a need to analyze the fluctuation trends. Since almost every person in the world is a part of the transportation industry, and the transportation industry influences a country's economy, it is essential to observe the behavior of price drifts in gasoline and diesel price [9].

Volatility in gasoline and diesel prices may impact market participants, tax revenues, international oil market price fluctuations, and vulnerable groups. While much past research has been done on price drifts concerning gasoline and diesel, limited studies have been done from artificial intelligence (AI) perspective.

Therefore, we are employing an AI-based technique for analyzing the drifts in gasoline and diesel prices, i.e., Bayesian Regularized Neural Network (BRNN). The strength of BRNNs lies in the fact that they are much advanced compared to traditional back-propagation nets [10]. They could also minimize the need for lengthy cross-validation [11]. The model's design, training, validation, and testing have been performed using accurate historical market data.

Gasoline and diesel prices indicate the growth level of the nation’s economy. Plenty of work was carried out using trends of the hefty data segregated on fuel prices using machine learning techniques. Considered datasets hold 13 parameters over the period and drifts are there due to the pandemic in addition. The lifestyle changed a lot due to the unprecedented events in the recent pasts and that have a great role in deciding the near future. We have used the neural network (NN)-based concepts that helped in building a higher accuracy model.

The main contributions of the current work are as follows:

- We are analyzing the US gasoline and diesel price drifts which will help the investors and policy decision-makers to provide an overview for the market during the crisis.
- Our cumulative work precisely indicates that NAR-NN serves the best purpose in the past historical data.
- We have also compared three training algorithms LM, SCG, and BR, based on the NN concept, and BR showed noticeable results from others.

Even though various models prevail in machine learning to analyze and predict. The NN-based structure outperforms better because of the three-layered structure with the meticulous hidden layer structure that makes the model so proficient in nature. BR is robust in nature in comparison with regular back-propagation networks. Also, it achieves better correlation, and the sum of square errors is minimal.

The rest of the article is structured as follows: Sect. 2 lists materials and methods incorporating the related works subsection and the proposed work's methodology. In Sect. 3, we have discussed the experimental analysis, including the dataset and the evaluation parameters in detail. Section 4 highlights the results in the form of observations, graphs, and comparative analysis. Finally, Sect. 5 concludes this article.

## Materials and Methods

This section is divided into two subsections. In the first subsection, we are highlighting the related research works done in the past, following which we have discussed our proposed approach.

### Related Works

Authors in [12] presented a study on detecting gasoline adulteration utilizing altered distillation curves and artificial neural networks (ANN). Their study was conducted to find the temperature and recovered volume simultaneously. Image processing was performed level metering, and gasoline and diesel were added to distillation curves for analyzing the effect of additives. The ANN predicted the volume percentage of contaminants in super gasoline, and statistical analysis vouched for the model’s efficiency.

Authors in [13] studied the COVID-19 pandemic's impact on Turkey's gasoline consumption. A unique data set of daily data from 2014 to 2020 was employed for their study. The performance was forecasted using the Autoregressive integrated moving average (ARIMA) model, and evaluation was performed before and after the outbreak. The best fit models seem to fail in the pandemic situation; hence, forecasting improves adding volatility. Their study asserts that policies targeting volatility may effectively reduce the adverse impacts on revenues, vulnerable groups, and market participants.

Authors in [14] investigated gasoline compression ignition (CGI) in diesel engines utilizing computational fluid dynamics. A single-cylinder engine experiment was considered for validating the results. The model captured the combustion performance, which was analyzed using an estimation of energy breakdown and emissions. Their study further asserts that injection strategy and injector nozzle configuration lead to a better fuel stratification profile, increasing the engine and emissions performance. A comparison has been carried out between diesel and CGI in the same operating conditions and hardware. Their study manifests that simultaneous optimization of engine and fuel can efficiently overcome the combustion performance trade-off.

Authors in [15] presented a study on the price elasticity of demand for diesel, gasoline, hybrid, and battery-electric cars. Their study aimed to retrieve direct and cross-demand market response functions confined to Norway and was for the automobile powertrains and their energy carriers. The carbon dioxide emissions from automobiles were found to be related to vehicles and energy prices. Their study was conducted using a discrete choice model on 1.8 million data points. An increase in the price of liquid fuel leads to a reduction in the carbon dioxide emission rate.

Authors in [16] conducted a household-level survey to estimate gasoline price reforms and consumption behavior in Saudi Arabia. A total of 1800 responses were obtained. Their analysis manifests that the January 2016 price hike may be attributed to the 20% drop in gasoline usage among users who utilized octane 91-type gasoline. Octane 95 consumed 15% more gasoline, and the estimated demand elasticity decreased with education level. It was also found that income levels are connected with sophisticated consumption in advanced price periods.

Authors in [17] studied China's gasoline price concerning international crude oil price and regulation. Their analysis was conducted to determine the fluctuations and price regulation using a panel-asymmetric error correction model with daily panel data. The primary observation made is that the price response is symmetric concerning industry but asymmetric concerning several refiners. China's gasoline cost is equal to fuel oil value changes; however, unevenly corresponding to value guidelines, prompting mutilations in the oil market and cost reaction elements.

Authors in [18] performed a study on gasoline and diesel demand for 118 countries based on the fuel prices, economic growth, and demand for gasoline and diesel. The data incorporates 36 countries for over 39 years, i.e., 1978–2016. The panel addresses problems such as cross-sectional dependence, nonstationary, and heterogeneity. Their study manifested that Organization for Economic Co-operation and Development (OECD) gasoline cost elasticity is − 0.7, whereas the OECD diesel cost elasticity is − 0.35. For non-OECD, diesel price elasticity is almost similar to that of gasoline.

Authors in [19] studied the demand for gasoline and diesel in Europe. The Autoregressive-Distributed Lag (ARDL) model has been used to measure the short-run and long-run costs along with income elasticities for diesel and gasoline demands. The data spans from 1978 to 2013 and observed that elasticity estimates vary across countries. The short-run and long-run elasticities seem significantly elastic concerning their price equivalents. Therefore, if the fuel charge is meant to decrease emissions by the price hike, the charge must rise higher than income. Their study appeals for a stringent fuel tax policy.

Authors in [20] recommended an AI and information-driven approach to analyze Saudi Arabia's energy markets. Their model GANNATS is a combination of data mining (DM), genetic algorithm (GA), and ANN along with time-series (TS) analysis, and the design, training, validation, and testing of this model have been done on actual historical market data. Experiment analysis manifested that the model performs efficiently. Cross-validation determined that Saudi Arabia's gasoline mandate went down by 2.5% in 2017. A screening analysis identified the factors leading to gasoline demands. Their model enhanced traditional econometric models and also increased the efficiency of gasoline demand forecasting.

Authors in [21] performed a TS analysis and forthcoming trends prognosis on gasoline price in China based on oil taxation and costing techniques. Their work explored a statistical relationship between crude oil costs and gasoline prices, supported by TS and error correction models. A projection of the prices for the years 2019–2050 has been estimated. It is seen that asymmetric responsiveness and the threshold effect exist within the Chinese oil pricing policy. There is also a lag of at least a month in the gasoline price adjustment. Their study asserted that gasoline prices will be affected by the crude oil price increase in the short run, and in the long run, gasoline prices would be affected by the crude oil price decrease.

Authors in [47] introduced a way to foresee new COVID-19 cases by utilizing hybridized approach between machine learning (ML), adaptive neuro-fuzzy inference system (ANFIS) and upgraded beetle antennae search (BAS) swarm intelligence metaheuristics.

Table 1 provides a summarized analysis of some of the existing related works deploying computational and ML methods.

**Table 1:** Related work analysis with limitations

References	Proposed work	Methodology	Limitation
[12]	Detected gasoline adulteration	Modified distillation curves and ANN	Distillation may have issues with azeotropic mixtures, energy consumption, and chemical reactions. ANNs may be hardware-dependent and may require multiple trials and errors
[13]	Impact of COVID-19 pandemic on gasoline consumption for Turkey	ARIMA model	Forecasting extreme values may be difficult with ARIMA
[14]	CGI in diesel engines	Computational fluid dynamics	Computationally intensive, multiple errors due to simplified boundary conditions
[19]	Demands for gasoline and diesel in Europe	ARDL model	Possibility of Multicollinearity, lag length may be more in smaller samples
[20]	Analyzed energy markets in Saudi Arabia	GA, ANN, DM along with time-series (TS)	Computationally expensive, time-consuming
[22]	Gasoline cost in China	TS analysis with future trends projection	Generalization issues, identifying the correct model may be challenging

Table 2 provides a related work analysis of various existing works with their research outcomes and methodologies used.

**Table 2:** Related work analysis with results

Reference	Proposed work	Methodology	Results
[20]	AI and information-driven prescient model to dissect and measure gasoline interest of Saudi Arabia	Artificial neural network (ANN) + Genetic algorithm (GA) + data mining (DM) for TS analysis	Saudi gasoline requests lowered in 2017 by 2.5% from its 2016 usage
[23]	Shown a hybrid multi-specialist model of a petroleum market populated without anyone else intrigued (profit-maximizing) specialists	Multi-agent simulations (MAS) + Geographical Information System (GIS)	The proposed model was found to beat a multi-specialist model that did not represent significant framework conduct, e.g., the developments and support of shoppers
[24]	Surveyed reasons that there are no single pointers driving oil costs and there is a need to distinguish the significant input factors for oil cost expectations	Literature review	Multi-layered perceptron NN is most broadly utilized by specialists for value anticipating
[25]	Proposed a novel hybrid expectation technique by consolidating a mysterious organization TS examination and AI calculations	Data fluctuation network (DFN) + several AI algorithms	The anticipating execution of the proposed DFN-AI model is magnificent regardless of random sample selection, sample frequency, or sample structural breaks, demonstrating its heartiness and dependability
[26]	The study pointed toward exploring petrol costs by drawing consideration on the nonlinear conduct and instability structure	LSTAR–LST–GARCH + LSTAR–LST–GARCH–NN	The models with fractional combination and asymmetric force GARCH gave huge increases contrasted with straightforward GARCH models
[27]	Utilized a recurrent NN model to conjecture the non-directly shifting fuel costs in significant metropolitan urban communities of India	Recurrent Neural network model	From the perceptions, it tends to be found that the anticipated qualities are exceptionally near the genuine costs. The exactness of the model is above 90% at next-day costs or more than 80% for a one-week figure

### The Proposed Approach

Clustering is a crucial information examination technique. It is broadly utilized for pattern recognition, feature extraction, etc. As an unsupervised classification strategy, clustering distinguishes some natural designs present in a set of items dependent on a similarity measure [22]. The self-organizing map (SOM) is an incredible strategy for data visualization, clustering, etc. It has been utilized effectively for high dimensionality and intricacy where customary strategies may frequently be deficient. To investigate information construction and to find cluster limits from the SOM, one usual methodology is to address the SOM's information by representation strategies. Existing strategies introduced various parts of the data learned by the SOM, yet information geography, which is available in the SOM's information, is enormously underutilized [28].

**Fig. 1:**
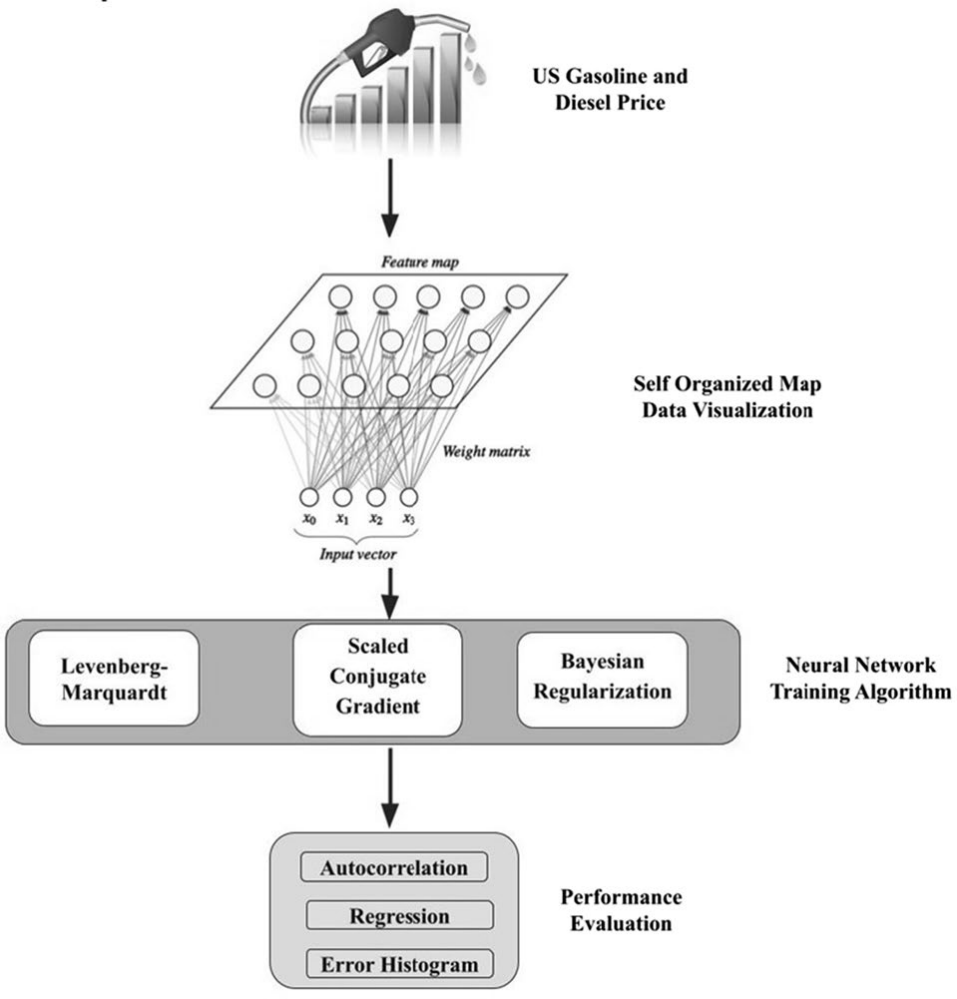
The proposed approach

Analyzing the current pricing and fitting the appropriate model that yields better performance metrics is carried out in this work. Figure 1 illustrates the workflow of the current analysis.

The SOM calculates the Euclidean distance for the input pattern *y* to every neuron *l* and finds the winning neuron, denoted neuron *t*, with prototype *B*_*t*_, utilizing the nearest-neighbor rule. The winning node is known as the excitation center. For all the input vectors closest to *B*_*t*_, upgrade all the prototype vectors by the Kohonen learning rule [29]. Assuming:

1 $$B_{t} \left( {m + 1} \right) \, = \, B_{t} \left( m \right) \, + \, \mu \left( m \right) \, \left[ {y_{m} - \, B_{t} \left( m \right)} \right]$$

2 $$B_{l} \left( {m \, + \, 1} \right) \, = \, B_{l} \left( m \right) \, + \, \mu \left( m \right) \, i_{lt} \left( m \right) \, [y_{m} {-} \, B_{t} \left( m \right), \, l \, = \, 1, \, . \, . \, ., \, L$$

where *µ(m)* persuades the Robbins–Monro criteria and *i*_*lt*_*(m)* is the excitation response or neighbor function, which tells neuron t when *B*_*t*_ is the excitation center. If *i*_*lt*_*(m)* takes *δ*_*lt*_, (2) minimizes the SCL. *i*_*lt*_*(m)* could be considered as a function that decreases with the increasing distance between *B*_*l*_ and *B*_*n*_, and typically as the Gaussian function in (3):

3 $$i_{lt} (m) = i_{0} e^{{\frac{{ - ||B_{l} - B_{t} ||^{2} }}{{\beta^{2} \left( m \right)}}}}$$

from the where the constant *i*_*0*_ > 0, *β(m)* is a decreasing function of m with a popular choice, $\beta \left( m \right) = \beta_{0} e^{{ - \tfrac{m}{Y}}}$, *β*_*0*_ being a positive constant and *ϒ*, a time constant [30].

**Fig. 2:**
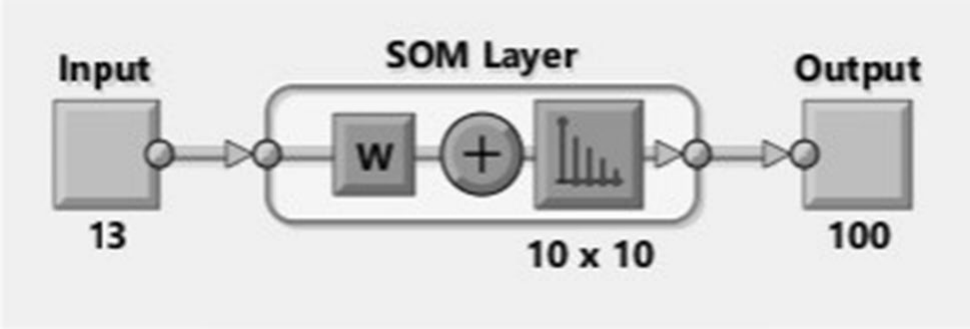
SOM-layered architecture

The Gaussian function is organically more sensible than the rectangular. The SOM utilizing the Gaussian area merges much rapidly than utilizing the rectangular one [31]. Figure 2 shows that the number of variables considered is 13 and 10 × 10 layers used to map the input data visualization.

In the most recent couple of years, much exploration has been coordinated at comprehension and foreseeing what is to come. Albeit conventional measurable TS strategies perform well, many have inalienable constraints. In the first place, without skill, it is feasible to wrongly indicate the utilitarian structure relating the autonomous and ward factors and neglect to make the essential information changes. Second, anomalies can prompt one-sided assessments of model boundaries [32]. Moreover, TS models are regularly direct and accordingly may not catch nonlinear conduct. Many have contended that neural organizations can survive or, in any event, be less dependent upon these constraints [33]. These cases will be surveyed in no time. Some conventional measurable TS strategies have intrinsic restrictions because of how the models are assessed. When numerous sorts of conventional factual TS models are assessed, human association and assessment are required. Likewise, numerous customary factual strategies do not adapt steadily as new information shows up; all things considered; they should be re-assessed intermittently. It has been guaranteed that neural organizations can likewise overcome these issues [34]. The TS gauges dependent on neural organizations were contrasted and estimates from conventional measurable TS techniques (counting remarkable smoothing and Box-Jenkins) and a judgment-based strategy [35]. The neural organization model improved conventional factual and human judgment strategies when gauging quarterly and month-to-month information. Notwithstanding, the neural organization model and conventional models were equivalent to the yearly information.

A NAR-NN can anticipate a TS from that series of past qualities $X\left( {s - 1} \right), \, X\left( {s - 2} \right), \ldots ,X\left( {s - t} \right)$ called feedback delay, with *t* being the time defer boundary. The network is made and prepared in an open circle, utilizing the genuine objective qualities as a reaction and ensuring more superior quality being exceptionally near the genuine number in preparing. In preparing, the network is changed over into a shut circle, and the anticipated qualities are utilized to supply new reaction contributions to the network. A NAR applied to TS anticipating depict a discrete, nonlinear autoregressive model that can be written in this structure (4):

4 $$X_{t} = \, g \, \left( {X_{s - 1} , \, X_{s - 2} , \ldots , \, X_{s - t} } \right) \, + \, \Upsilon_{s} .$$

**Fig. 3:**
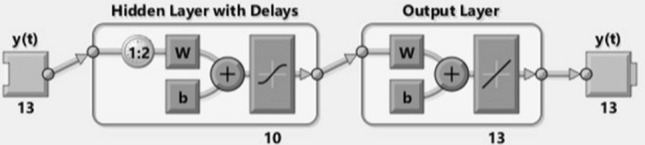
NN layered architecture

The function g(.) is obscure ahead of time. The training of the NN is pointed toward approximating the function by methods for optimizing the network weights and neuron bias. Along these lines, a NAR model is characterized decisively by a condition of the sort (5)

5 $$X_{s} \, = \, \beta_{0} \, + \, \sum\nolimits_{k = 1}^{j} \beta_{{\text{k}}} \varphi {( }\sum\limits_{a = 1}^{b} {\alpha_{ak} X_{s - a} } { + }\alpha_{0k} {) + }\Upsilon_{s} ,$$

where b is the number of entries, *j* is the number of hidden layers with activation function $\varphi$, and $\alpha_{ak}$ is the parameter corresponding to the weight of the connection among the input layer a and the hidden layer k, $\beta_{k}$ is the weight of the connection among the hidden layer k and the output unit, $\alpha_{0k}$ and $\beta_{0}$ are the constants that correspond, respectively, to the hidden layer k and the output unit [36]. Figure 3 indicates the 13 inputs with 10 hidden layers in the NAR-NN model.

As indicated by Bayesian method [53], the values A and B (parameters) values at the minimum point of objective function E are settled by posterior probability and Eq. (9) is derived:

6 $$A = \frac{Y}{{2S_{W} }}.$$

Here, $Y$ is the effective weight of sample network parameter, $S_{W}$ is the sum of the square of the network weight.

7 $$B = \frac{N - Y}{{2S_{D} }}.$$

Here, $N$ is the no. of connection right.

8 $$Y = m - 2A.tr(H)^{ - 1}$$

9 $$H = B\nabla^{2} S_{D} + A\nabla^{2} S_{W} .$$

Here, $H$ is the Hessian matrix of the objective function. The Bayesian NN training can adjust the size of $Y$and make it optimal.

Levenberg–Marquardt's (LM) calculation is a reiterative procedure that finds the lowest function to be communicated as the number of squares of nonlinear functions. It has become a benchmark strategy for nonlinear least-squares issues and could be considered a blend of steepest descent and the Gauss–Newton technique. When the current arrangement is far from the right one, the calculation carries on like a steepest descent technique: moderate yet ensured to 1 meet [37].

Conjugate Gradient strategies are a class of vital techniques for limiting smooth functions, mainly when the measurement is massive [38]. They are viewed as conjugate direction or gradient deflection strategies between steepest descent and Newton's strategy. Their chief benefit is that they do not need the capacity of any grids as in Newton's strategy or as in quasi-Newton techniques, and they are intended to unite quicker than the steepest descent technique [39].

Bayesian regularized artificial neural networks (BRANNs) are a powerful approach than typical back-propagation nets and could diminish or dispense with the requirement for extensive cross-validation.

Bayesian regularization (BR) is a numerical cycle that changes over a nonlinear regression into an “all-around presented” measurable issue using an edge regression. The benefit of BRANNs is hearty, and the validation interaction, for example, back-propagation, is pointless. These networks answer various issues that emerge in QSAR, demonstrating, like the decision of model, the strength of the model, decision of validation set, size of validation exertion, and network engineering improvement. They are hard for excess training since proof strategies give a target Bayesian rule to halting preparation. They are also hard to overfit, because the BRANN figures and prepares on various viable network boundaries or weights, adequately killing those most certainly not applicable. This successful number is generally significantly more modest than the weights in a standard completely associated back-propagation neural net. Automatic relevance determination (ARD) of the info factors can be utilized with brands. Furthermore, this permits the network to “gauge” the significance of each info. The ARD technique guarantees that unessential or exceptionally associated files utilized in the displaying are dismissed just as showing, which are the main factors for demonstrating the movement information [10].

## Experimental Analysis

This section will provide details about the dataset considered for the experiment, followed by detailed parameters considered for performance evaluation.

### Dataset and Data Visualization

For experimentation, we have considered the dataset [40]. The dataset consists of 1361 weekly gasoline and diesel prices in the U.S. in $/gallon from January 1995 to January 2021. The details of the dataset are presented in Table 3 as follows:

**Table 3:** Dataset description

Criteria	Details
A1	Weekly US all grades all formulations retail gasoline prices (Dollars per Gallon)
A2	Weekly US all grades conventional retail gasoline prices (Dollars per Gallon)
A3	Weekly US all grades reformulated retail gasoline prices (Dollars per Gallon)
R1	Weekly US regular all formulations retail gasoline prices (Dollars per Gallon)
R2	Weekly US regular conventional retail gasoline prices (Dollars per Gallon)
R3	Weekly US regular reformulated retail gasoline prices (Dollars per Gallon)
M1	Weekly US midgrade all formulations retail gasoline prices (Dollars per Gallon)
M2	Weekly US midgrade conventional retail gasoline prices (Dollars per Gallon)
M3	Weekly US midgrade reformulated retail gasoline prices (Dollars per Gallon)
P1	Weekly US premium all formulations retail gasoline prices (Dollars per Gallon)
P2	Weekly US premium conventional retail gasoline prices (Dollars per Gallon)
P3	Weekly US premium reformulated retail gasoline prices (Dollars per Gallon)
D1	Weekly US No 2 diesel retail prices (Dollars per Gallon)

**Fig. 4:**
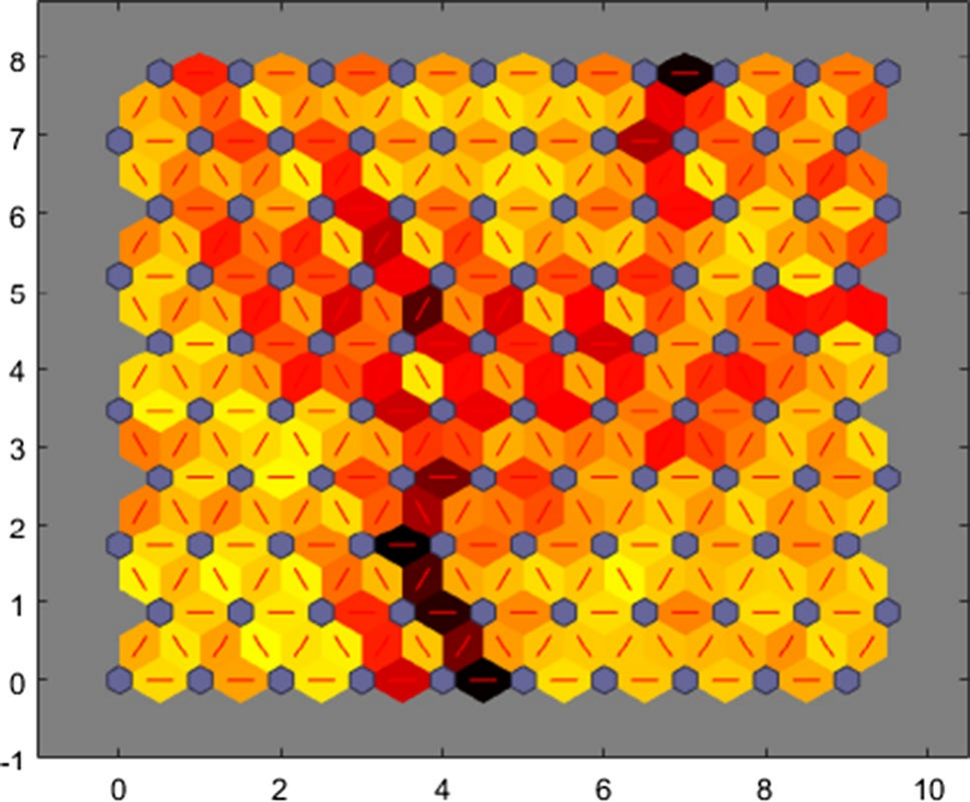
NN training SOM neighbor weigh distances

In Fig. 4, neurons are represented in blue color. Neighbor neurons are connected via a red line, and it narrates about distances. The higher intensity illustrates that those distances are large and lighter intensity another way.

**Fig. 5:**
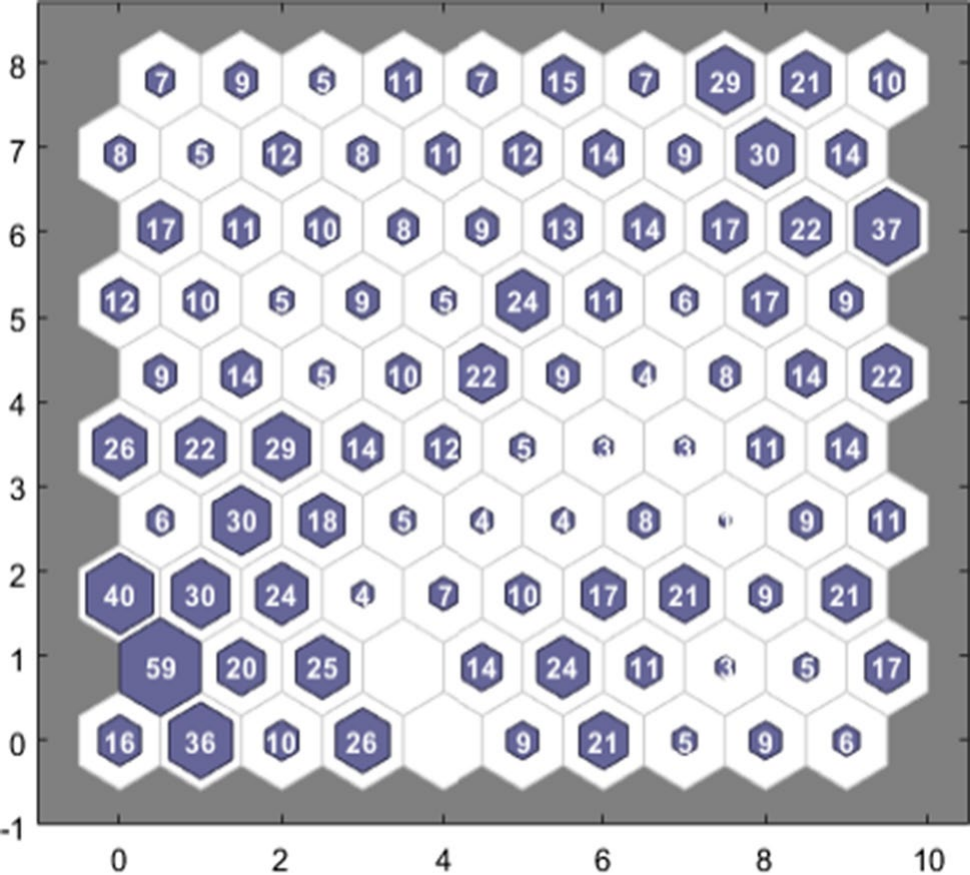
NN training SOM hits

SOM is exhibited in a hexagonal structure. The topology is illustrated in 10 × 10 grids with 100 neurons. In this SOM visualization, a maximum of 59 input vectors is prevailing, shown in the maximum number of hits. Figure 5 illustrates the association between data points and neurons. A commonly accepted version is that data should be distributed evenly to get better results. The data concentration is relatively high on the upper right, and lower left neurons, but it is distributed.

**Fig. 6:**
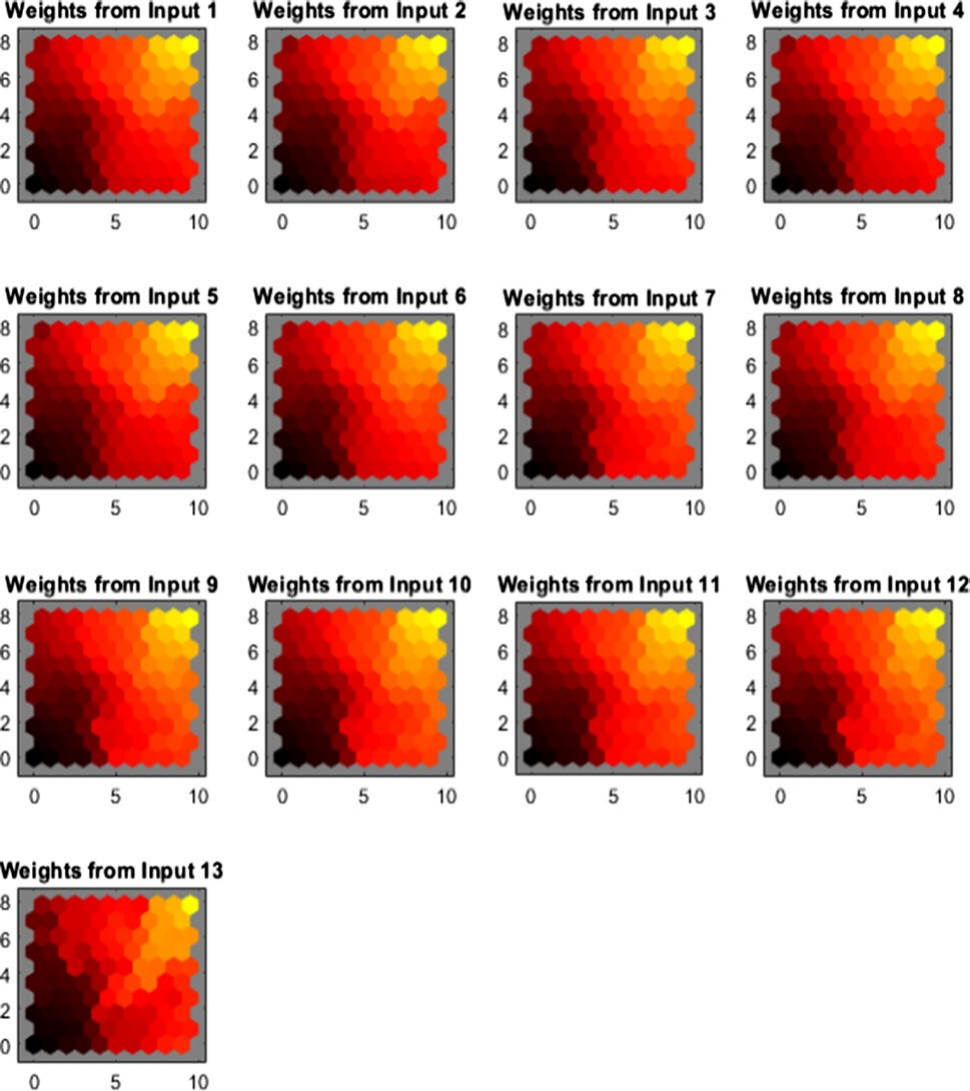
SOM weigh planes

SOM weight panes provide the weight of the individual attributes considered in the analysis and the case of our work. The exact weight of 13 attributes is depicted in Fig. 6 as the usual intensity of colors indicates the relationship between data and neurons. The pattern of figures can infer similarities and traits. In the present work input, 1–12 are similar, indicating the petrol products have similar data used. Still, diesel's last input is exhibiting varied values that are visible by the arrangement of cells.

## Results and Discussion

### ***NN***—TS Analysis

**Fig. 7:**
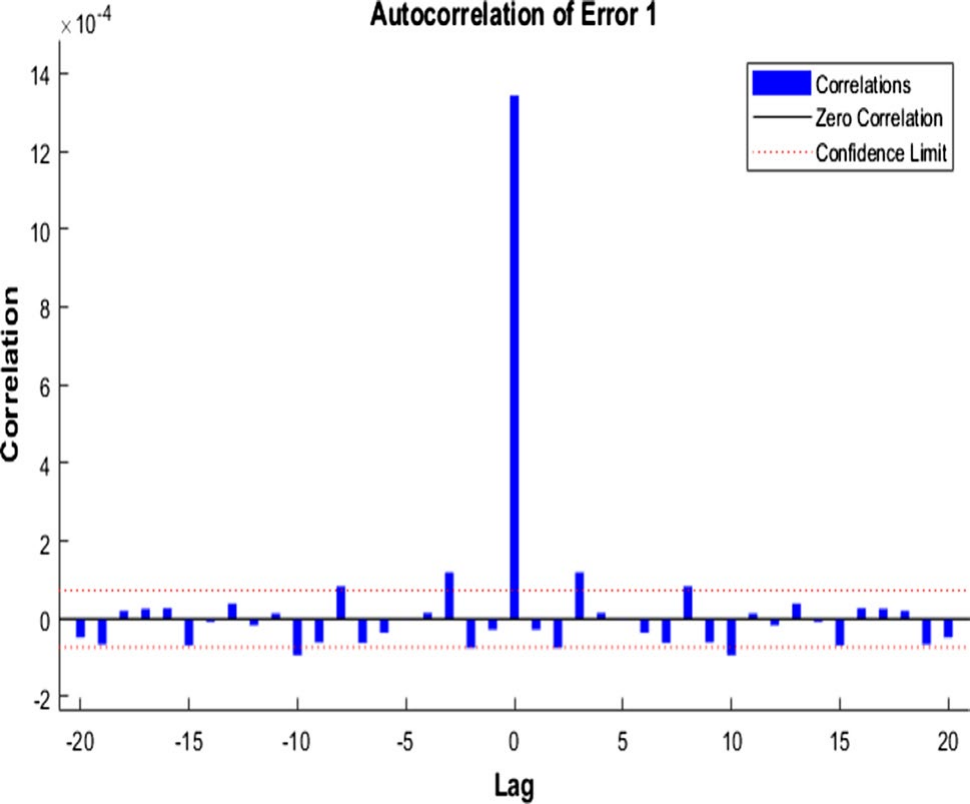
Autocorrelation of Error 1—LM

**Fig. 8:**
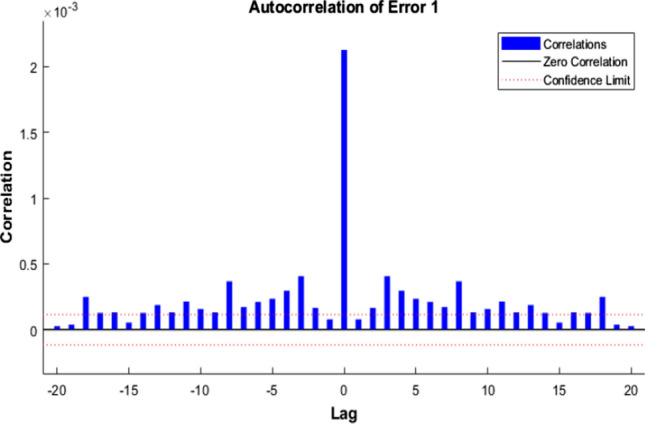
Autocorrelation of Error 1—SCG

**Fig. 9:**
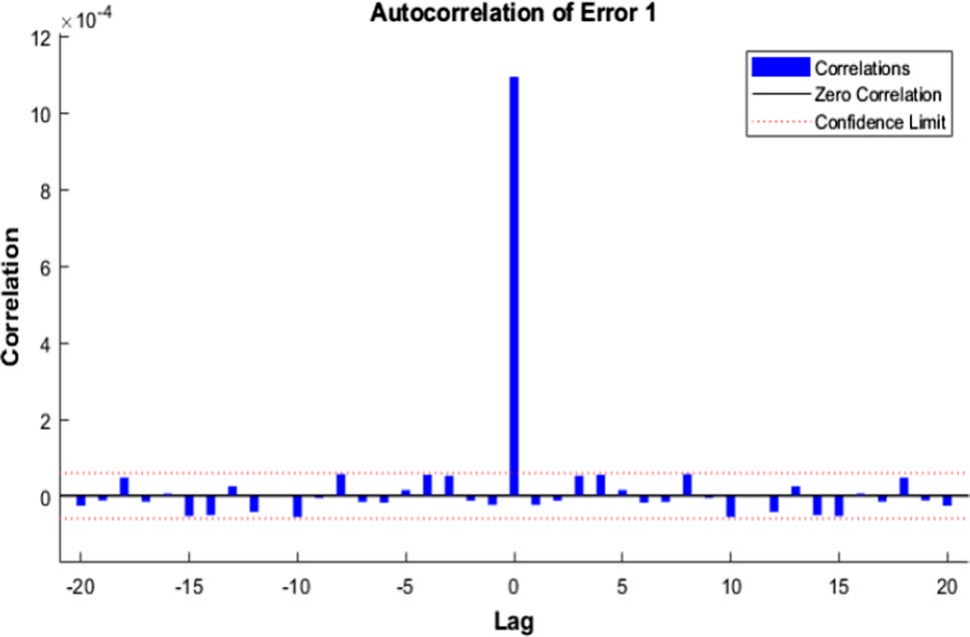
Autocorrelation of Error 1—BR

Figures 7, 8, 9 represent the ACE function that gives insight into the relationship between prediction error and time.

The ideal model should have only one non-zero value and zero lag—confidence level indicated by a dotted red line around 1.

After comparing the plots obtained by the three different algorithms, it is evident that BR has perfect results. We can observe only one non-zero value at the zero lag, and a confidence limit of 95% is visualized. The rest of the approaches are not that perfect in comparison with the ideal model perspective.

**Fig. 10:**
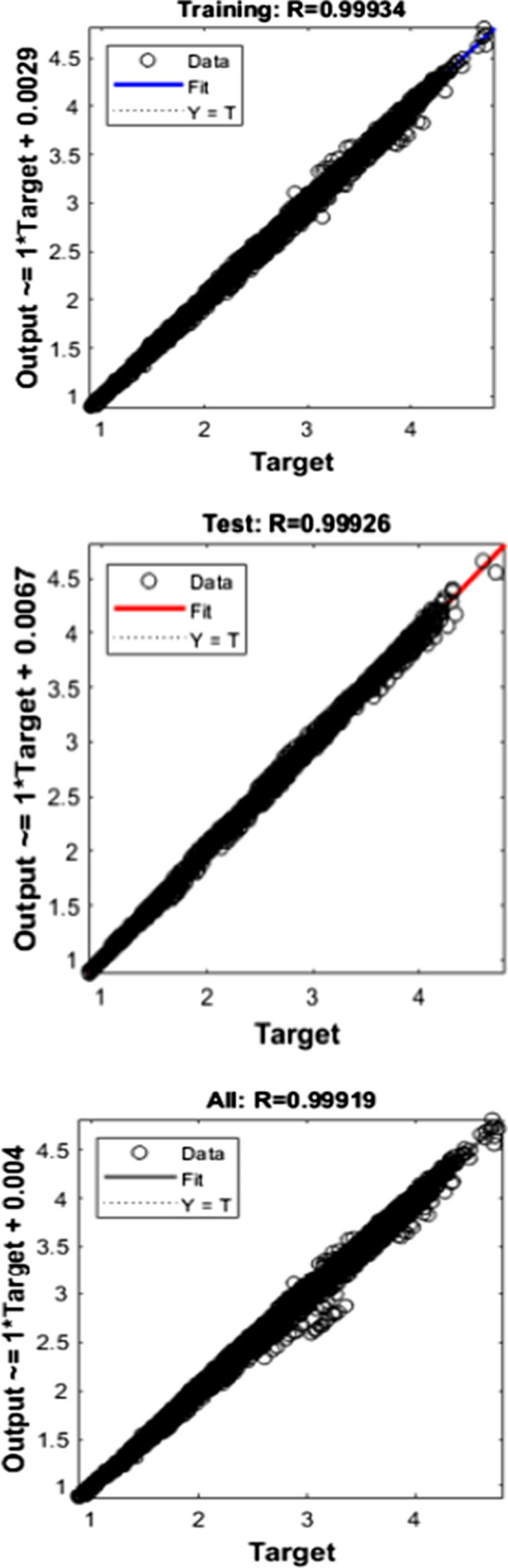
Regression—LM

**Fig. 11:**
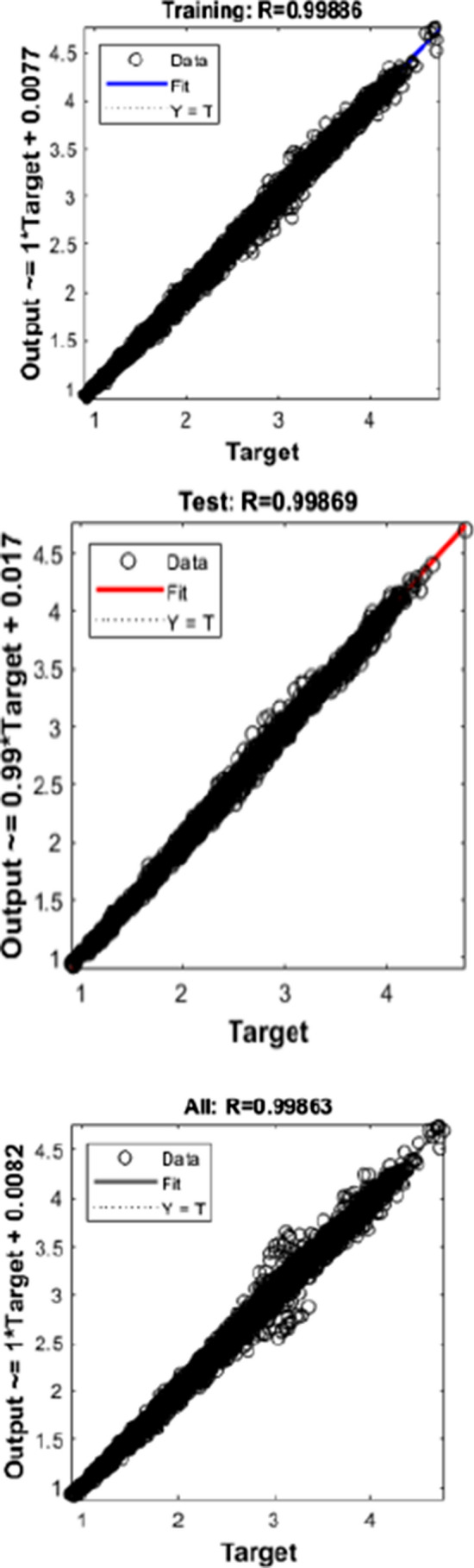
Regression—SCG

**Fig. 12:**
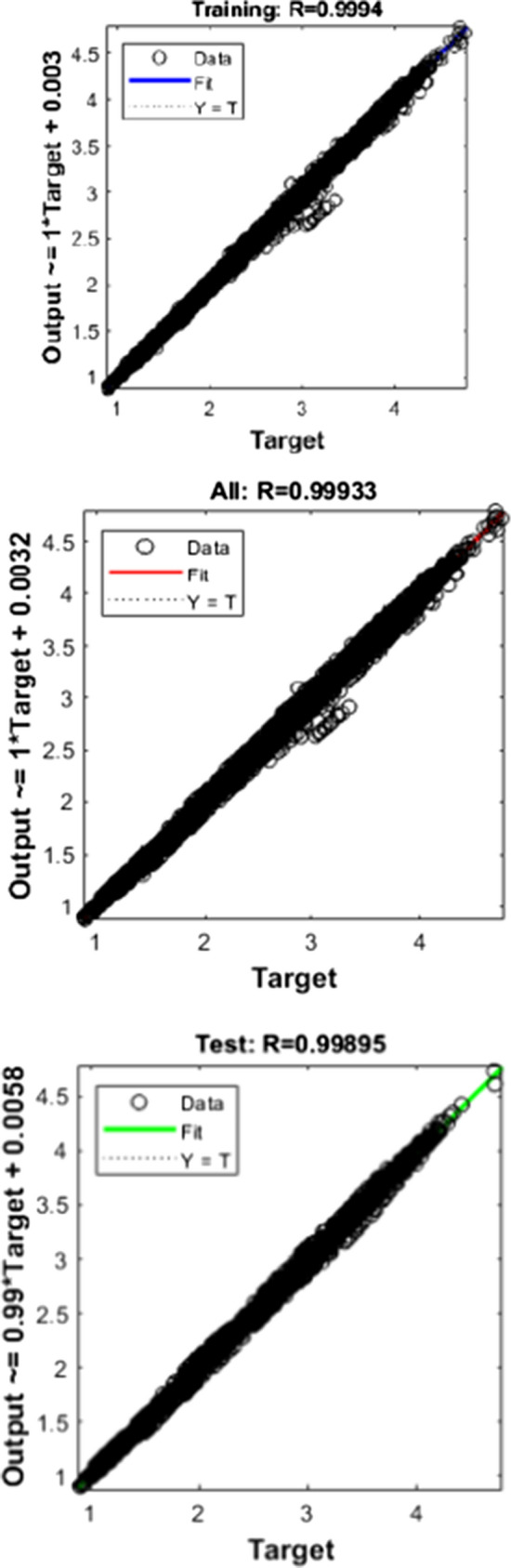
Regression—BR

Figures 10, 11, 12 depict the regression graph for three different algorithms, and it is having a perfect fit for the dataset considered.

An ideal interpretation for the best fit is that the data should fall along a 45° line.

In this, it is evident that the R-value is more significant than 0.99 in all the cases.

**Fig. 13:**
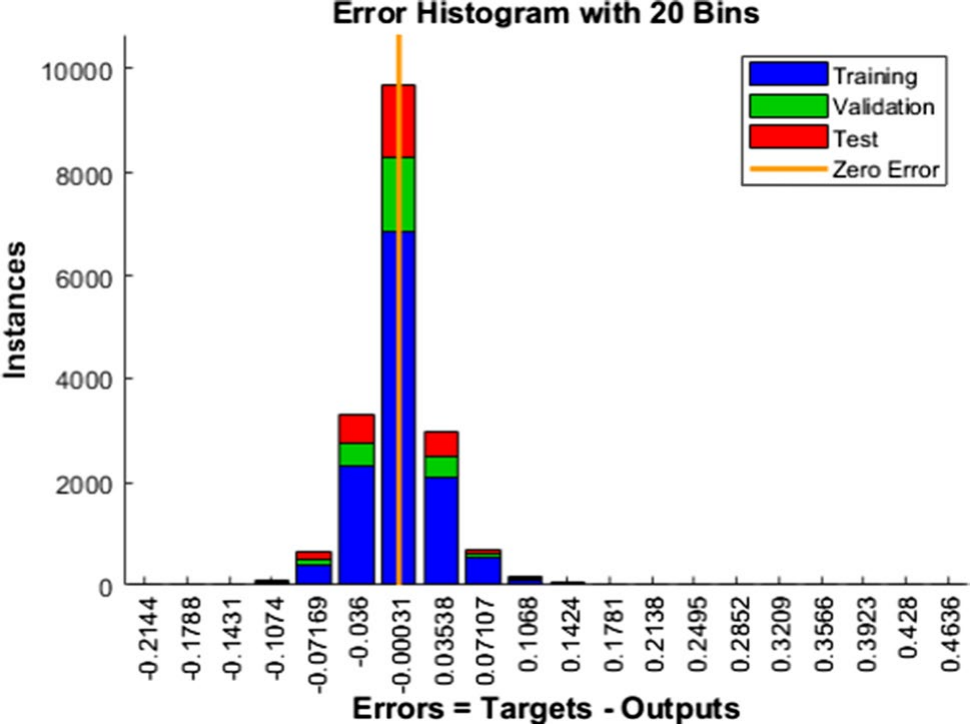
Error Histogram—LM

**Fig. 14:**
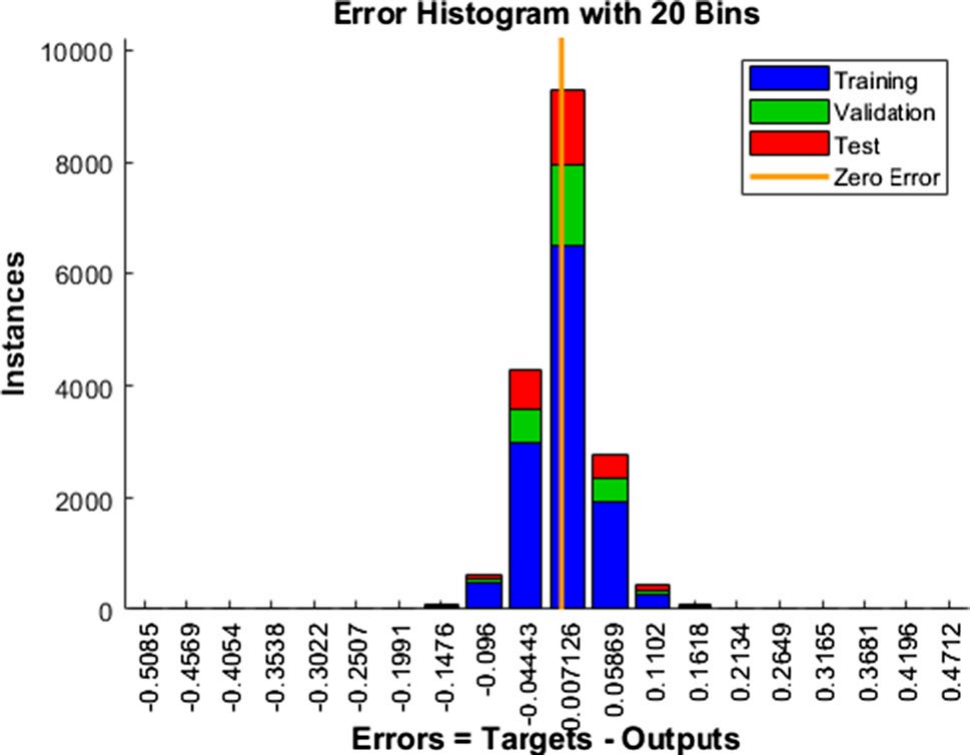
Error Histogram – SCG

**Fig. 15:**
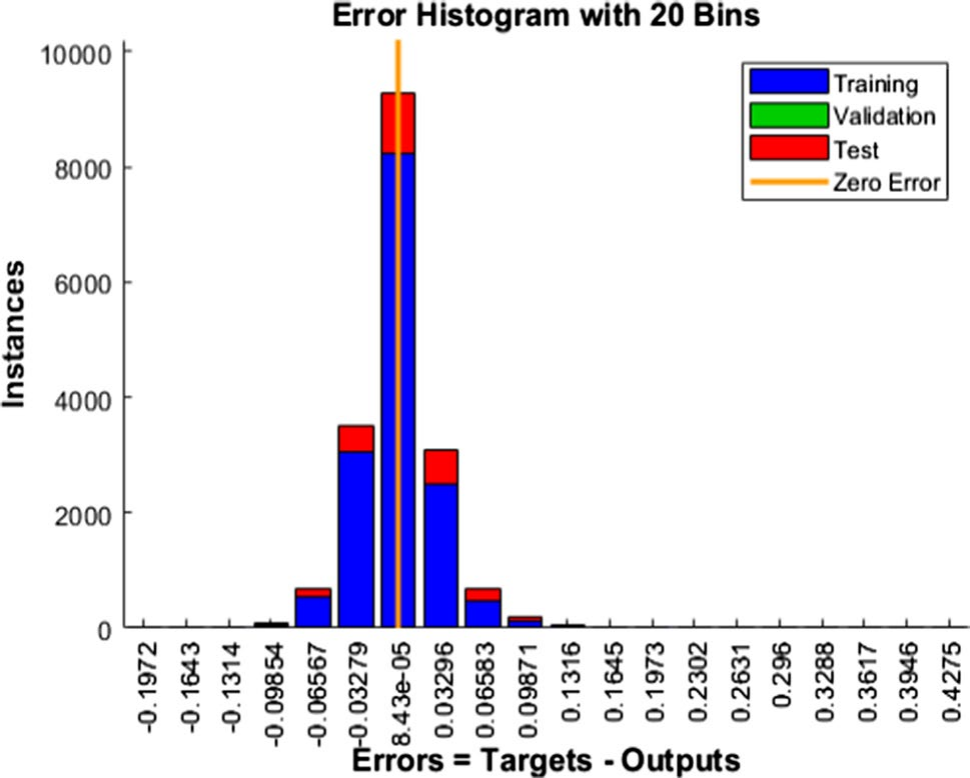
Error Histogram—BR

Figures 13, 14, 15 graphical represent the error histogram is the additional aid that helps gauge the network's performance.

Blue, green, and red colors in the bar represent the insight about training, validation, and test data utilized.

The vertical yellow color line indicates the zero-error stuff. In our work, LM error falls from − 0.1074 to + 0.1424, SCG error falls from − 0.1476 to 0.1618, and similarly, BR error falls from − 0.09854 to 0.09871. From these mentioned data points, it is evident that BR exhibits minimum errors, and its performance is commendable.

### Performance Evaluation

For experimentation purposes, we have considered MATLAB R2020a.

Tables 4, 5, 6 provide the MSE and R-value. In the present work, the target value taken for training is 12385, validation and testing are 2654 for all the three methods, namely LM, SCG, and BR. In NAR, the no. of hidden layers, lags, and neurons are the principle hyperparameters, which impact the accuracy of the outcomes. It is ideal to think about all hyperparameters simultaneously; in any case, it expands the processing time [52].

**Table 4:** Training with different approaches

Approach/Parameter	MSE	*R*
LM	0.00104448	0.99934
SCG	0.00184333	0.998864
BR	0.00970452	0.999397

**Table 5:** Validation with different approaches

Approach/Parameter	MSE	*R*
LM	0.00252317	0.998444
SCG	0.00397423	0.997493
BR	0	0

**Table 6:** Testing with different approaches

Approach/Parameter	MSE	*R*
LM	0.00124506	0.999259
SCG	0.00202854	0.998694
BR	0.00168114	0.998948

For better visibility and understanding, Tables 4, 5, 6 results have been plotted with the box and whisker approach.

**Fig. 16:**
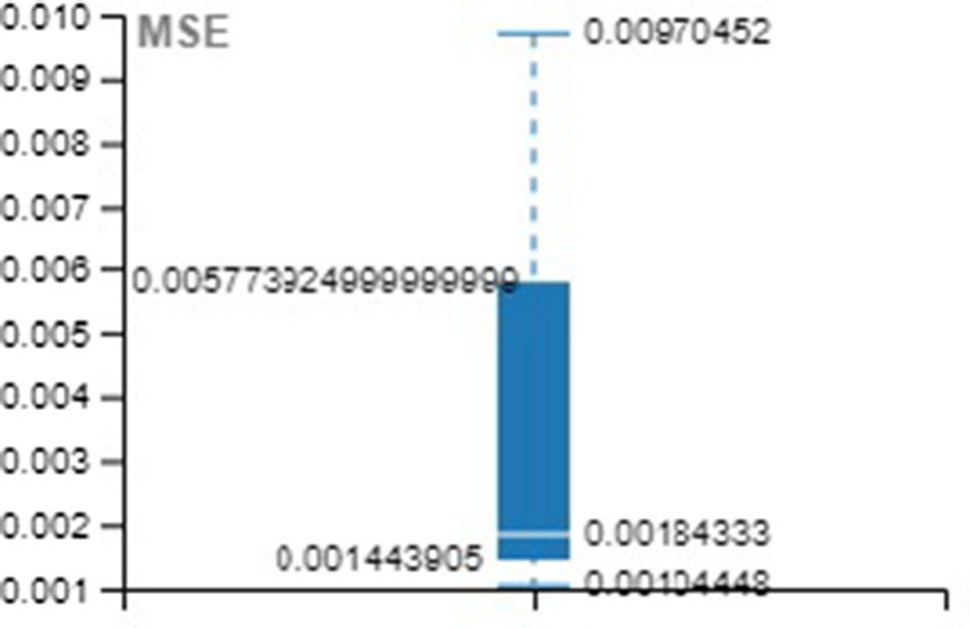
Box plot for training (MSE)

**Fig. 17:**
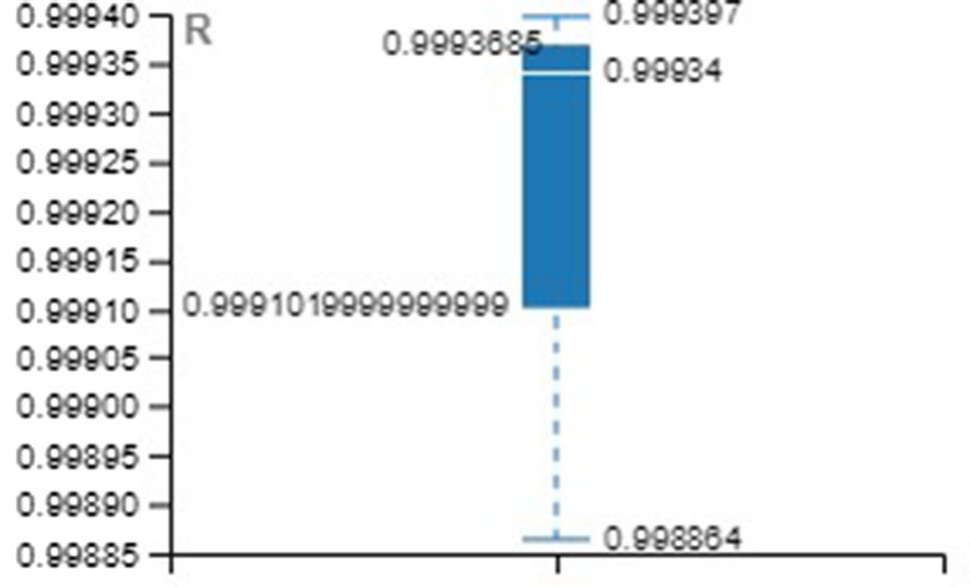
Box plot for training (R)

MSE and R values of the various approach over the training set are illustrated in Figs. 16 and 17.

**Fig. 18:**
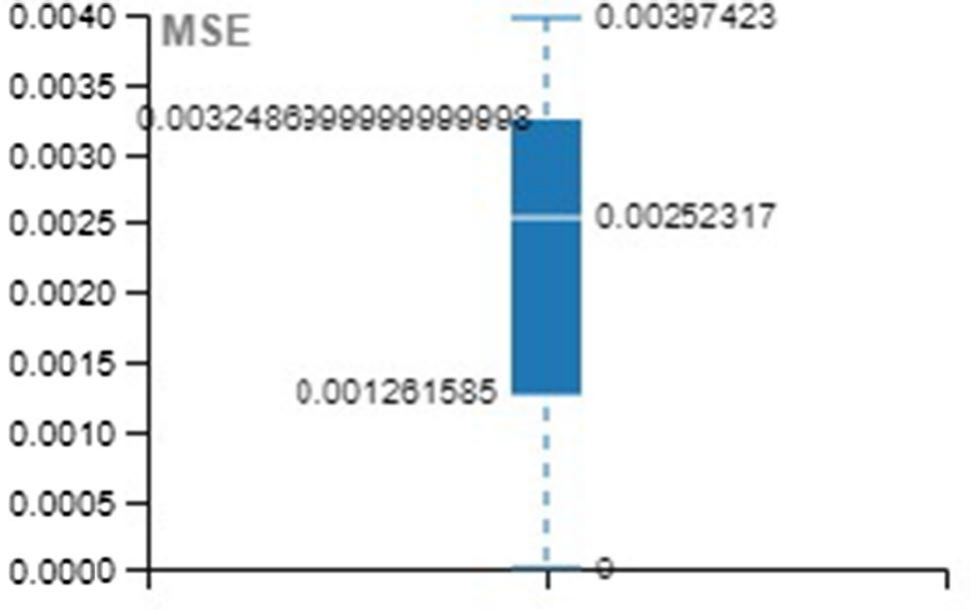
Box plot for validation (MSE)

**Fig. 19:**
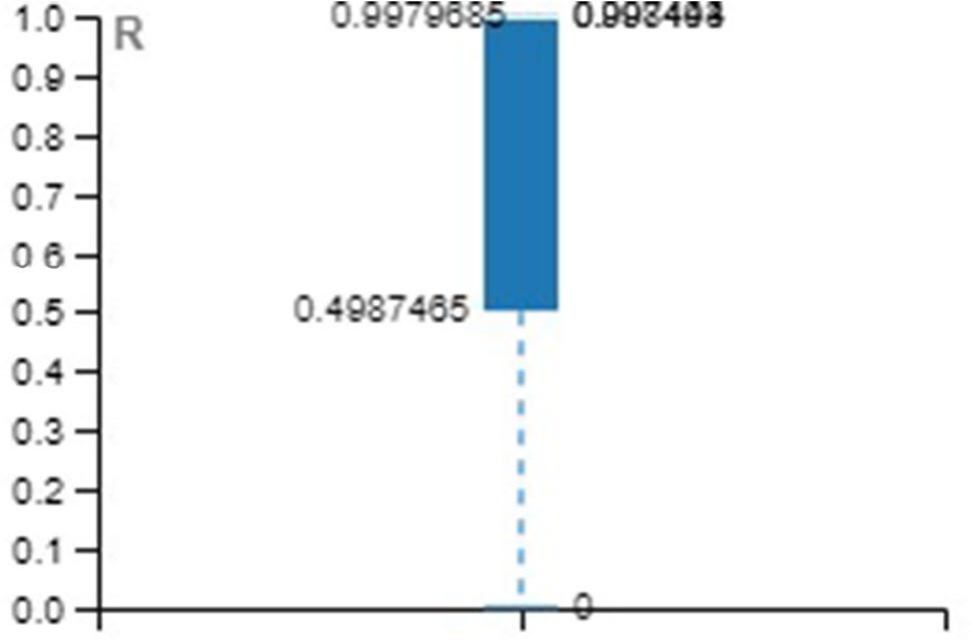
Box plot for validation (R)

MSE and R values of the various approach over the validation set are illustrated in Figs. 18 and 19.

**Fig. 20:**
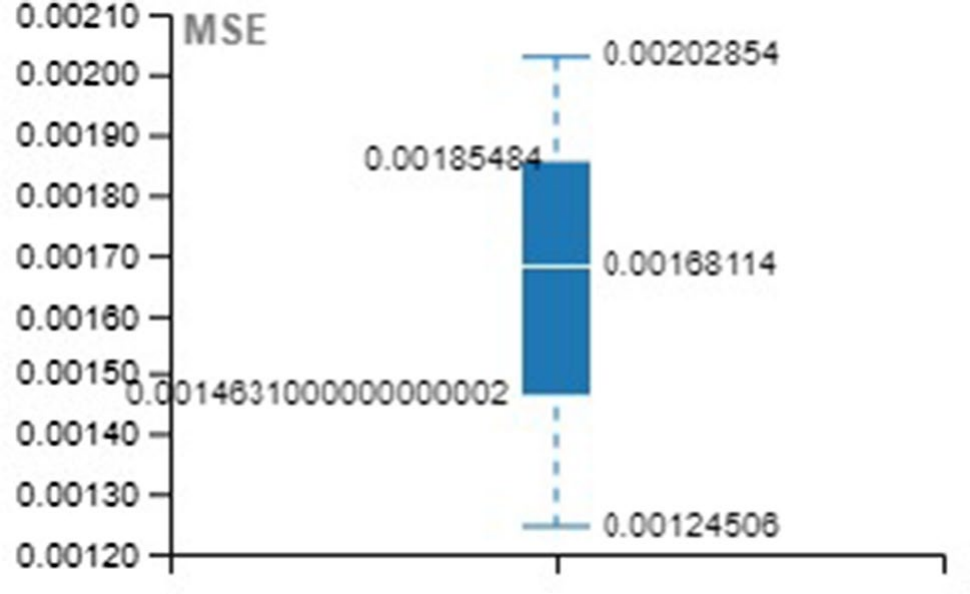
Box plot for testing (MSE)

**Fig. 21:**
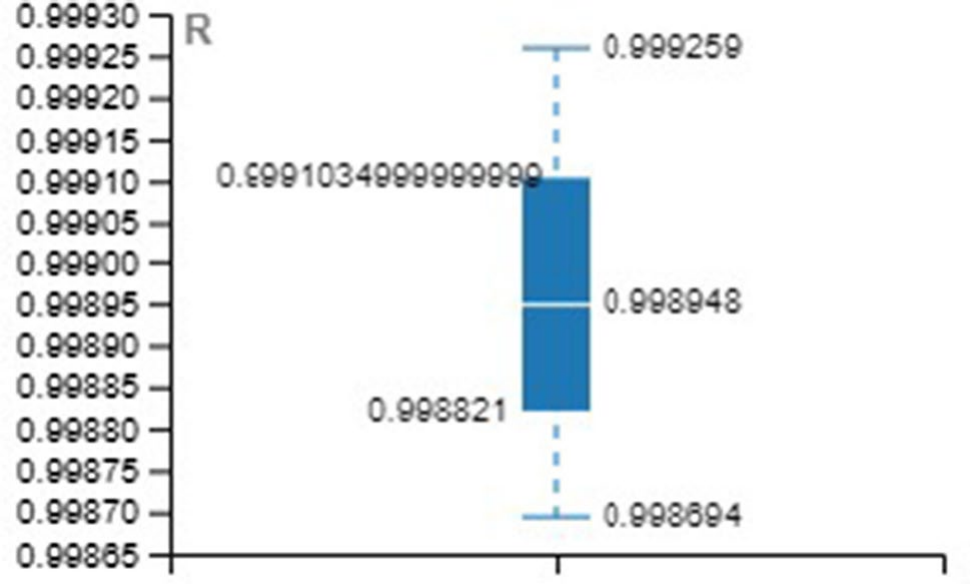
Box plot for testing (R)

MSE and *R* values of the various approach over the testing set are illustrated in Figs. 20 and 21.

LM converges slowly when the numbers of parameters are more than 10 and, in this model, the considered parameters are 13. Similarly, in the case of SCG, function minimization takes n cycle, and, in that way, it becomes expensive in nature. Experimentally, our proposed BR approach outperformed the SCG and LM models.

Table 7 provides a comparative analysis of various existing works with our proposed work.

**Table 7:** Comparative analysis

Reference	Proposed work	Methodology	Results
[41]	Estimation of the uranium price	BR-based multilayer feed-forward neural network	Cumulative distribution function = 0.0720, Average mean relative error (AMRE) = 0.0533
[42]	Thermal error modeling for the feed drive system of computer numerical control (CNC) machine tool	Bayesian Neural Network (BNN)	Their proposed model attains a 71% reduction in the thermally induced error
[43]	Forecasting of Gold prices	BRNN	Mean percentage error ~ 1%
[44]	Calibration of an impedance probe for soil moisture measurement	BNN	For generalized calibration, RMSE < 0.04 m3m-3
[45]	Fast supersymmetric predictions	BNN	Average percentage errors < 3.34%
[46]	Evaluation of agricultural water distribution	HBNs	The mean absolute percentage error is 17.31%, coefficient of determination is 0.96
[48]	Suggested Multivariate Long Short-Term Memory (MLSTM) with Mahalanobis and Z-score transformations	MLSTM + Z-score	Feature selection and outlier elimination data were given an RMSE of 0.212 and an R2 score of 0.954
[49]	Introduced Empirical mode decomposition-Support Vector Machine (EMD-SVM) model	EMD-SVM	The performance of the EMD-SVM is outperformed than the individual ANN and SVM with 2.2777 as MAE, 4.9518 as MAPE and 2.5470 as RMSE
[50]	Suggested sequence-to-sequence LSTM and a WaveNet-inspired Temporal CNN (TCN) for forecasting Australian petrol prices	TCN	The TCN model gave the best result with a 1.38% MAPE over a 60-day forecast window utilizing the test information
[51]	A deep belief network (DBN) made of restricted Boltzmann machines (RBM) for pre-train and a layer of supervised back-propagation (BP) for calibrating gold price is proposed	DBM + RBM + BP	DBN model has the best performance, with the lowest RMSE of 0.0557, 0.0064 as MAPE, and 0.0460 as MAE, and the highest Dstat of 0.5738 with ten hidden layers
Our proposed work	Analyzed U.S Gasoline and Diesel Price Drifts	BRNN	LM error falls from -0.1074 to + 0.1424, SCG error falls from -0.1476 to 0.1618 and similarly, BR error falls from -0.09854 to 0.09871

## Conclusion

Many industries’ growth and future are visualized with the help of prevailing historical data. Based on the analysis, corrective and preventive measures need to bring the industry's growth to the next level. In a few cases, like the stock market, gold prices, fuel prices require careful intervention for the investors and the country’s economic reliability purpose. GDP of the country and price of commodities highly plunged due to acute variation of the prices. The NAR-NN is used for the gasoline and diesel dataset. The ACE and error histogram illustrates that BRNN is outperforming, thus indicating the optimal forecast approach that could be utilized. BR outperforms well in comparison with the average back-propagation nets. In the proposed approach, LM error falls from − 0.1074 to + 0.1424, SCG error falls from − 0.1476 to 0.1618, and similarly, BR falls from − 0.09854 to 0.09871. It is also observed in the ACE plot that only one non-zero value at the zero lag with a confidence limit of 95% and with minimal errors. The research results show that BR exhibits minimum errors, and its performance is higher than other approaches. The ACE plot can observe only one non-zero value at the zero lag, confidence limit of 95%, and minimal errors. Strategies like lockdown for a day in the week to protect nature could be instilled. Similarly, it helps make various policy decisions based on economic, political, business, and vertical. Incorporating other related attributes on various demography like income, pollution, etc., will provide great insight into the country's growth and be considered the extension of the proposed work. In the future, we will also try to work in the direction of computational complexity analysis for better results.

## Data Availability

The dataset used in this research is openly accessible via: https://www.eia.gov/dnav/pet/pet_pri_gnd_dcus_nus_a.htm
